# Atrial and ventricular arrhythmia predictors with electrocardiographic parameters in myocardial infarction with non-obstructive coronary artery disease (MINOCA)

**DOI:** 10.5830/CVJA-2022-045

**Published:** 2022-09-08

**Authors:** Serkan Asil, Muhammet Geneş, Salim Yaşar, Serdar Fırtına, Suat Görmel, Erkan Yıldırım, Yalçın Gökoğlan, Hatice Tolunay, Barış Buğan, Ayşe Saatçi Yaşar, Murat Çelik, Uygar Çağdaş Yüksel

**Affiliations:** Department of Cardiology, Gülhane Training and Research Hospital, Ankara, Turkey

**Keywords:** arrhythmia, atrial fibrillation, electrocardiography, MINOCA, ventricular tachycardia

## Abstract

**Background:**

The clinical importance and recognition of myocardial infarction with non-obstructive coronary artery disease (MINOCA) is increasing. Nevertheless, no studies are investigating the risk of atrial fibrillation and ventricular arrhythmia in MINOCA patients. This study aimed to determine the risk of arrhythmia with electrocardiographic predictors in MINOCA patients.

**Methods:**

In this study, patients diagnosed with MINOCA and stable out-patients without significant lesions in their coronary arteries were compared. Morphology–voltage–Pwave duration electrocardiography (MPV ECG) score was used to determine atrial arrhythmia risk. QT interval and QT dispersion Tpeak–Tend time and Tpeak–Tend/QT interval were used to determine ventricular arrhythmia risk.

**Results:**

A total of 155 patients were included in our study. Seventy-seven of these patients were in the MINOCA group. There was no statistically significant difference between the two groups in MPV ECG score (1.95 ± 1.03 vs 1.68 ± 1.14, p = 0.128). P-wave voltage, P-wave morphology and P-wave duration, which are components of the MPV ECG score, were not statistically significantly different. The QRS complex duration (90.21 ± 14.87 vs 82.99 ± 21.59 ms, p = 0.017), ST interval (271.95 ± 45.91 vs 302.31 ± 38.40 ms, p < 0.001), corrected QT interval (438.17 ± 43.80 vs 421.41 ± 28.39, p = 0.005) and QT dispersion (60.75 ± 22.77 vs 34.19 ± 12.95, p < 0.001) were statistically significantly higher in the MINOCA group. The Tpeak–Tend (89.53 ± 32.16 vs 65.22 ± 18.11, p < 0.001), Tpeak–Tend/QT interval (0.2306 ± 0.0813 vs 0.1676 ± 0.0470, p < 0.001) and Tpeak–Tend/corrected QT interval (0.2043 ± 0.6997 vs 0.1551 ± 0.4310, p < 0.001) ratios were also significantly higher in patients with MINOCA.

**Conclusion:**

In the MINOCA patients, there was no increase in the risk of atrial fibrillation based on ECG predictors. However, it was shown that there could be a significant increase in the risk of ventricular arrhythmia. We believe this study could be helpful for specific recommendations concerning duration of hospitalisation and follow up in MINOCA patients.

Myocardial infarction with non-obstructive coronary arteries (MINOCA) describes patients presenting with clinical features of an acute myocardial infarct (MI) without obstructive coronary artery disease (CAD).^1^,^2^ The primary determinant of the clinic for MINOCA is ischaemic CAD without an obvious over-50% obstruction in the coronary arteries.^1^ Furthermore, the reason for elevation of non-ischaemic cardiac enzymes must be excluded.

The pathophysiology of MINOCA is multifactorial and similar to atherosclerosis risk factors.^1^ Atherosclerotic plaque disruption is the most common underlying cause in intravascular coronary imaging-based and angiographic-based studies.^3^,^4^ Other possible underlying causes are coronary vasospasm, microvascular dysfunction, coronary thrombus-embolism, spontaneous coronary dissections and myocardial oxygen supply/demand mismatch.^1^

The underlying cause determines the prognosis of patients with MINOCA. Most research suggests that MINOCA patients had better outcomes than patients with MI with obstructed CAD.^5^ However, this conclusion is not shared by all researchers. Furthermore, some of them reported that MINOCA patients have an increased risk of future adverse events such as MI with obstructive CAD.^6^,^7^

While the arrhythmic prognosis of patients with MI with obstructive CAD is well documented, there is little data concerning MINOCA, even when the arrhythmic prognosis seems to be relatively good. Because of the underlying pathophysiological mechanism, an increased risk of atrial and ventricular arrhythmias may be predicted in MINOCA patients. However, the number of studies showing an increase in arrhythmia in the literature is quite limited.

As a result of the lack of data concerning arrhythmia, there are limited specific recommendations concerning duration of hospitalisation and follow up for this specific setting. For example, atrial fibrillation (AF) is the most common arrhythmia in daily clinical practice. Identifying MINOCA patients at risk for AF using simple, non-invasive and widely accessible electrocardiographic (ECG) markers would be extremely useful for clinicians in determining which patients should be monitored more closely and for more extended periods.^8^ On the other hand, ventricular arrhythmias are the most critical, life-threatening arrhythmias, and necessary recommendations and precautions can be applied by pre-detection of the risk utilising ECG.^9^

This study aimed to determine whether MINOCA patients may be at increased risk of AF and ventricular arrhythmias based on readily available ECG measurements, compared to stable patients without significant lesions in their coronary arteries.

## Methods

In this retrospective cohort study, patients who were admitted to the emergency department with chest pain and high cardiac troponin levels between January 2020 and September 2021, who were diagnosed as MINOCA patients, were identified and included. The control group included patients who were scheduled to undergo coronary angiography under out-patient clinic conditions but did not have significant lesions (< 50% stenosis) in their coronary arteries.

The coronary angiography of MINOCA patients was evaluated by at least two invasive cardiologists’ visual evaluation and quantitative angiography (QCA) measurements. The pathophysiological mechanisms of MINOCA were investigated from the coronary angiography images of patients. One of the most important limitations of our study is that we could not give precise information about the underlying pathophysiology of MINOCA since we could not use routine intravascular ultrasound (IVUS) and optical coherence tomography (OCT).

The literature defines vulnerable plaque morphology angiographically in studies performed before IVUS and OCT. Based on these studies, we determined that irregular borders or intraluminal lucency, haziness and slowing of the flow rate in the lesion area are characteristic of vulnerable plaque.^10^,^11^ Suspected clinical vasospastic angina was diagnosed by acetylcholine provocation test, but a routine provocation test was not performed to detect epicardial coronary vasospasm for all patients.

The inclusion criteria in both groups were age above 18 years, patients who were scheduled to have angiography as out-patients and who were not found to have significant coronary stenosis. Exclusion criteria in both groups were defined as patients whose file records and ECGs could not be accessed from the hospital archive and data system; those with atrial fibrillation, atrial flutter, multifocal–unifocal atrial tachycardia, atrial extrasystole, ventricular tachycardia, ventricular extrasystole and ventricular conduction delay in the admission ECG or medical history of the patients; patients with unstable cardiac status (in unstable clinical conditions such as unstable angina and decompensated heart failure, repolarisation and depolarisation abnormalities are frequently observed in the ECG); patients with organ failure (chronic renal failure, cirrhosis, chronic obstructive lung disease, stroke and dementia), malignancy and infectious status.

All procedures were performed as per the Declaration of Helsinki. The local ethics committee approved the study (University of Health Sciences Gulhane Training and Research Hospital Ethics Committee decision number 2021-362, date: 21.10.2021).

Following a 10-minute rest period, 12-lead ECGs were acquired in a supine position using a commercially available ECG machine (GE Healthcare, MAC 2000) with 10 mm/mV amplitude and 25 mm/s rates and standard lead placements. The ECG records of the patients were taken before coronary angiography in the control group and on the day of admission when cardiac enzymes were elevated in the MINOCA patients to eliminate bias. Each ECG lead was measured with at least five consecutive beats as an average, depending on heart rate. ECG images were 10-fold amplified and measured with Image J software. Two blinded cardiologists who had no information about the patients examined each image.

We investigated P waves after determining that the isoelectric interval existed and that the P waves did not fuse with the preceding QRS complex or T wave. PR intervals were calculated from the onset of the P wave to the beginning of the QRS complex, and PR segments were calculated from the end of the P wave to the beginning of the QRS complex. P-wave duration was assessed in leads II, III and aVF of the 12 surface ECG leads. The voltage of the P wave in lead I was used to determine the wave’s peak or nadir to the isoelectric line. P-wave duration ≥ 120 ms was considered as prolongation.

Partial inter-atrial conduction block was defined as P-wave duration ≥ 120 ms. Advanced inter-atrial block was defined as prolongation and a biphasic (±) morphology in the inferior leads. The morphology–voltage–P‐wave duration (MPV) ECG AF risk score is a simple and effective method that can predict the risk of AF, utilising ECG, which has only recently been used in the research.^12^,^13^ Three P‐wave variables formed the MPV score: P-wave morphology in the inferior leads, the voltage of the P wave and duration in lead 1.^12^

The QRS complex, which is a composite of the Q, R and S waves, represents ventricular depolarisation and is measured from the end of the PR interval (or the beginning of the Q wave) to the end of the S wave. The QT interval, measured from the beginning of the QRS complex to the end of the T wave, was calculated using the Bazett formula: QTc = QT (R–R interval). QT dispersion was measured within each lead by comparing the longest (QTmax) and shortest (QTmin) QT intervals.

The Tpeak–Tend interval was defined as the interval between the T wave’s peak and end. Tpeak–Tend interval measurements were taken using precordial leads. These measurements were used to calculate the Tpeak–Tend/QT and Tpeak–Tend/QTc ratios. Studies use QT interval, QT dispersion Tpeak–Tend time, and Tpeak–Tend/QT interval ratios to predict ventricular arrhythmia risk from ECG in many diseases and clinical conditions.^9^,^14^,^15^

All echocardiographic examinations were performed using the ultrasound imaging system with S4-2transducer (GE Healthcare, Vivid S70N). Apical four- and two-chamber images were acquired in the left lateral decubitus position, using the parasternal long and short axes. Two-dimensional images were used to determine the left atrial size, left ventricular (LV) end-systolic and LV end-diastolic diameters. Simpson’s biplane volume method was used to measure the ejection fraction. Diastolic function was evaluated following current guideline recommendations.^16^

## Statistical analysis

The SPSS program was used to perform all statistical analyses (version 20.0 for Windows, SPSS Inc, Chicago, IL). The data were compared in terms of the groups and evaluated by a normal distribution of the Shapiro–Wilk test and QQ plots. Continuous variables with normal distribution are expressed as mean ± standard deviation, non-normal distribution is expressed as median and interquartile range (IQR), and categorical variables are expressed as percentages when appropriate. The student’s t-test was used to analyse continuous variables with a normal distribution. The paired samples t-test and Mann–Whitney U-test compared numerical variables between the two groups. The chi-squared test was used to compare categorical variables between the two groups. A p-value of < 0.05 was considered statistically significant.

## Results

A total of 155 patients were included in our study. While 77 of these patients were in the MINOCA group, 78 patients were in the control group. In the MINOCA patients, a total of 41 (53.24%) had plaque disruption, 21 (27.27%) had microvascular dysfunction and slow flow, four had (5.19%) vasospasm and three (3.89%) had spontaneous coronary dissection. Conventional imaging could not determine the diagnosis in eight (10.38%) patients.

There were no statistically significant differences between the groups concerning gender, age, diabetes mellitus, hypertension, previous CAD and echocardiographic parameters. C-reactive protein (CRP) level (21.38 ± 43.87 vs 4.06 ± 2.60 mg/l, p < 0.001) and white blood cell (WBC) count (10.09 ± 3.70 × 103 vs 7.95 ± 5.08 × 103 cells/μl, p = 0.003), which are inflammatory markers in laboratory examinations, were statistically significantly higher in the MINOCA group. Clinical characteristics, laboratory examinations and echocardiographic features of the patients with MINOCA and the control subjects are shown in Table 1.

**Table 1:** Baseline clinical, laboratory and echocardiographic characteristics

*Characteristics*	*MINOCA (n=77)*	*Control (n=78)*	*p-value*
Age (years)	54.85 + 13.64	56.92 + 11.69	0.078
Women, n (%)	40 (51.9)	31 (39.7)	0.127
Diabetes mellitus, n (%)	18 (23.4)	24 (30.8)	0.300
Hypertension, n (%)	32 (41.6)	47 (60.3)	0.200
Previous coronary artery disease, n (%)	6 (7.8)	7 (9)	0.791
White blood cell count, X 103 cells/ul	10.09 + 3.70	7.95 + 5.08	0.003
C-reactive protein	21.38 + 43.87	4.06 + 2.60	< 0.001
Creatinin clearance (MDRD)	81.48 + 22.14	84.70 + 15.18	0.095
Left ventricular ejection fraction, %	58.05 + 8.47	60.07 + 5.10	0.073
Left ventricular end-diastolic diameter, mm	48.21 + 5.12	47.87 + 6.32	0.350
Left atrial diameter (parasternal long axis), mm	36.03 + 6.32	37.33 + 6.7	0.810
Left ventricular diastolic function, n (%)			
Normal	47 (61.03)	45 (57.69)	
Stage 1	28 (36.36)	30 (38.46)	
Stage 2	2 (2.59)	3 (3.84)	0.570
Stage 3	0	0	
Stage 4	0	0	

Numerical variables with normal distribution are shown as mean ± standard

deviation. Categorical variables shown as numbers (%).

MDRM: Modification of Diet in Renal Disease.

There was no statistically significant difference between the two groups in terms of MPV ECG score, a new and valid electrocardiographic predictor of atrial arrhythmia (1.95 ± 1.03 vs 1.68 ± 1.14, p = 0.128). P-wave voltage, P-wave morphology and P-wave duration, which are components of MVP ECG score, were not statistically significantly different. The total P-wave duration was significantly higher in the MINOCA group than in the control group (110.87 ± 17.78 vs 104.36 ± 19.10 ms, p = 0.030). In addition, the PR interval was found to be statistically significantly lower in the MINOCA group than in the control group (156.95 ± 22.99 vs 167.08 ± 30.33 ms, p = 0.021). Electrocardiographic variables used for prediction of atrial arrhythmia are shown in Table 2.

**Table 2:** Comparison of atrial arrhythmia predictors in electrocardiography

*Predictors*	*MINOCA (n=77)*	*Control (n=78)*	*p-value*
Heart rate, bpm	81 + 26.40	72 + 23.50	0.001
PR interval, ms	156.95 + 22.99	167.08 + 30.33	0.021
PR segment, ms	62.57 + 26.40	69.23 + 30.77	0.151
P-wave duration, ms (%)	110.87 + 17.78	104.36 + 19.10	0.030
< 120	41 (53.2)	47 (60.3)	
120-140	31 (40.3)	(35.9)	0.590
> 140	5 (6.5)	3 (3.8)	
P-wave voltage, mV (%)			
> 0.20	28 (36.4)	(33.3)	0.827
0.10-0.20	45 (58.4)	(62.8)	
< 0.10	4 (5.2)	3 (3.8)	
P-wave morphology, n (%)			
No inter-atrial block	29 (37.7)	(48.7)	0.284
Partial inter-atrial block	42 (54.5)	37 (47.4)	
Advanced inter-atrial block	6 (7.8)	3 (3.8)	
MVP ECG score	1.95 + 1.03	1.68 + 1.14	0.128
Low (0-2), n (%)	51 (66.2)	54 (69.2)	
Intermediate (3-4), n (%)	25 (32.5)	24 (20.8)	0.577
High (5-6), n (%)	1 (1.3)	0 (0)	

Numerical variables with normal distribution are shown as mean ± standard

deviation. Categorical variables shown as numbers (%).

MVP ECG: morphology–voltage–P‐wave duration electrocardiography.

The QRS complex duration (90.21 ± 14.87 vs 82.99 ± 21.59 ms, p = 0.017), ST interval (271.95 ± 45.91 vs 302.31 ± 38.40 ms, p < 0.001), corrected QT interval (438.17 ± 43.80 vs 421.41 ± 28.39 ms, p = 0.005) and QT dispersion (60.75 ± 22.77 vs 34.19 ± 12.95, p < 0.001) were statistically significantly higher in the MINOCA group than in the control group. The Tpeak–Tend (89.53 ± 32.16 vs 65.22 ± 18.11, p < 0.001), Tpeak–Tend/QT interval (0.2306 ± 0.0813 vs 0.1676 ± 0.0470, p < 0.001) and Tpeak–Tend/corrected QT interval (0.2043 ± 0.6997 vs 0.1551 ± 0.4310, p < 0.001) ratios were also significantly higher in patients with MINOCA than in the control group. Electrocardiographic parameters used for prediction of ventricular arrhythmia are shown in Table 3.

**Table 3:** Comparison of ventricular arrhythmia predictors in electrocardiography

*Predictors*	*MINOCA (n (=77)*	*Control (n=78)*	*p-value*
QRS complex duration, ms	90.21 + 14.87	82.99 + 21.59	0.017
ST segment, ms	113.61 + 40.09	131.15 + 46.12	0.130
ST interval, ms	271.95 + 45.91	302.31 + 38.40	< 0.001
QT interval, ms	389.03 + 37.08	390.71 + 29.12	0.754
Corrected QT interval, ms	438.17 + 43.80	421.41 + 28.39	0.005
QT dispersion	60.75 + 22.77	34.19 + 12.95	<0.001
T peak - T end interval	89.53 + 32.16	65.22 + 18.11	< 0.001
Tpeak - T end interval/QT interval	0.2306 + 0.0813	0.1676 + 0.0470	< 0.001
interval/corrected interval	0.2043 + 0.6997	0.1551 + 0.4310	< 0.001

Numerical variables with normal distribution were shown as mean ± standard

deviation. Categorical variables are shown as numbers (%).

MVP ECG: morphology–voltage–P‐wave duration electrocardiography.

## Discussion

In this trial, our primary finding was that the risk of AF, as indicated by the MVP ECG score, was not statistically significantly higher in the MINOCA patient group compared to the control group. Furthermore, the corrected QT interval, QT dispersion, Tpeak–Tend, Tpeak–Tend/QT interval and Tpeak–Tend/ corrected QT interval ratios were statistically significantly higher in the MINOCA patients than in the control group.

Studies on the prognosis of MINOCA patients have shown variables results. The significant determinants of these results are that a single aetiology for MINOCA does not exist and there was a limited number of participants.^17^ However, current data and guidelines have reported that the prognosis might worsen, as with MI with obstructive CAD. In these studies, groups were generally compared with regard to mortality, angina frequency, recurrent MI and quality of life.^6^,^7^ Approximately 25% of patients with MINOCA will experience angina in the subsequent 12 months, similar to the frequency reported in patients with MI with obstructive CAD.^18^ In addition, the study reporting that the risk of arrhythmia may increase in MINOCA patients during hospitalisation or out-patient follow up is limited.

Biere et al. showed in their study that MINOCA patients with an ischaemic late gadolinium enhancement (LGE) pattern on cardiac magnetic resonance imaging (MRI) experience more frequent ventricular arrhythmias during hospitalisation than patients without LGE in cardiac MRI.^19^ There was no difference in the incidence of arrhythmia between the two groups at the end of the one-year follow up.^19^

AF is the most common cardiac arrhythmia. However, no studies show an increased incidence and risk of AF in MINOCA patients during and after hospitalisation. Although the underlying pathophysiological mechanisms can be very different in MINOCA patients, it can be expected that the frequency of AF may increase due to the resulting myocardial ischaemia, as with MI with obstructive CAD. Furthermore, it has been previously reported that AF may be expected more frequently in MINOCA patients than in patients with MI with obstructive CAD (14.7 vs 7.3%).^20^ However, an increased frequency of AF at follow up was not reported in the same study.^20^

In order to predict the risk of AF on the surface ECG, many studies have been conducted.^21^ Alexander et al. recently developed the MVP ECG score as a novel scoring method for AF risk prediction.^12^ The MVP ECG score, which incorporates P-wave characteristics such as P-wave duration, inter-atrial block and P-wave voltage, represents atrial electrical and structural remodelling.^12^ According to MVP ECG scores, Alexander et al. classified study populations as low, medium or high risk and reported that the risk of AF increased 2.4 times in those with high MVP ECG scores.^12^

In 2019, after the article by Alexander et al., a few more studies on MVP scores were published. Yang et al. showed that this score could predict AF recurrence after pulmonary vein isolation.^13^ In one of these, Kahyaoğlu et al. showed a correlation between left atrial dysfunction demonstrated by speckle tracking echocardiography and MVP risk score in patients with hypertension.^22^ In another study, Hayiroglu et al. reported that ischaemic stroke patients with higher MVP ECG scores had higher AF incidence in index hospitalisation.^23^ A longer and strict follow up of these patients may be beneficial.

As a result of our study, we found that according to the MVP ECG risk score of MINOCA patients, 55 (66.2%) were in the low-risk group, 25 patients (32.5) were in the intermediate-risk group and only one patient (1.2%) was in the high-risk group. We did not find an increased risk of AF in the MINOCA patients compared to that of the control group.

Several ECG markers related to increased risk of ventricular arrhythmias and sudden cardiac death have been proposed.^24^ In various clinical situations and especially in patients with MI and heart failure, many studies have shown that QT, corrected QT and QT dispersion, which are markers of ventricular repolarisation, are associated with an increased frequency of ventricular arrhythmic episodes and may predict sudden cardiac death.^9^,^25^-^29^ The QT interval is between the beginning and end of ventricular repolarisation.

The heart rate-corrected QT interval has been proposed as a more accurate QT measure because QT is typically altered by heart rate.^30^ Although the QT interval was not statistically significantly different between the groups in our study, the corrected QT interval was statistically significantly different between the groups. We attributed this to the fact that MINOCA patients were more tachycardic due to their intensive care unit admission and acute ischaemic cardiac condition.

Other recently proposed ECG markers of ventricular repolarisation, the Tpeak–Tend, Tpeak–Tend/QT interval ratio and Tpeak– Tend/corrected QT interval ratio have been shown to predict ventricular arrhythmic events and sudden cardiac death in many studies.^14^,^31^-^34^ Tpeak–Tend/corrected QT ratio is a new measure for predicting cardiac arrhythmias that incorporates both transmural (Tpeak–Tend) and spatial (QT) ventricular repolarisation dispersion.^14^,^35^ Transmural repolarisation is caused by the different action potential durations of epicardial cells, M cells and endocardial cells.^14^ Tpeak–Tend indicates transmural dispersion of repolarisation, and this pathophysiological mechanism is generally thought to represent an increased risk of re-entry arrhythmias.^36^

The risk and mechanism of ventricular arrhythmia in MINOCA patients have not been previously investigated. However, similar arrhythmia mechanisms can be expected in these patients as they have similar pathophysiology mechanisms as patients with MI with obstructed CAD. During the first 10 minutes after MI, ventricular tachycardia is due to re-entry within the ischaemic myocardium.^37^,^38^ This is caused by a minimal decrease in conduction velocity and delayed recovery of excitability in relatively large circuits.^38^ In the reperfusion period, K+, Na+, and Ca2+ imbalance occurs in intracellular and extracellular components of the myocardium and leads to dispersion of refractoriness, which forms the substrate for re-entry.^37^,^39^

In the subacute phase of MI, the primary mechanisms of ventricular arrhythmia are automaticity in surviving Purkinje fibres and triggered activity due to delayed after-depolarisation.^37^ In the chronic phase, the primary mechanism, electrically inactive scar tissue forms a focus around which re-entrant circuits form.^37^

As a result, the most crucial arrhythmia mechanism in all MI phases is seen as a re-entry. Therefore, we believe that ECG parameters showing re-entry states such as Tpeak–Tend and Tpeak– Tend/QT interval can be used to predict ventricular arrhythmia in MINOCA patients.

Our study has many limitations. First, this was a small, singlecentre study. The main limitation of our study was the retrospective cross-sectional design of the study. Another limitation is the visual assessment of coronary angiographic images and the lack of IVUS and OCT for plaque characterisation.

## Conclusion

This study is the first on MINOCA patients to evaluate atrial and ventricular arrhythmia risk in ECG parameters. While no increase was found in the risk of atrial arrhythmia in MINOCA patients, an increase was found in the parameters showing heterogeneity in ventricular repolarisation, which is associated with the risk of ventricular arrhythmia. We believe this study could be helpful for specific recommendations concerning duration of hospitalisation and follow up in MINOCA patients. 
